# Unconventional roles of chromatin remodelers and long non-coding RNAs in cell division

**DOI:** 10.1007/s00018-023-04949-8

**Published:** 2023-11-20

**Authors:** Yuri Prozzillo, Maria Virginia Santopietro, Giovanni Messina, Patrizio Dimitri

**Affiliations:** 1 Dipartimento di Biologia e Biotecnologie “Charles Darwin”, Sapienza Università di Roma, Rome, Italy; 2 Present Address: Universita degli Studi di Milano-Bicocca, Piazza dell’ Ateneo Nuovo, 1, 20126 Milano, Italy

**Keywords:** Chromatin remodeling, Mitosis, Cytokinesis, Spindle, Midbody (MB)

## Abstract

The aim of this review article is to focus on the unconventional roles of epigenetic players (chromatin remodelers and long non-coding RNAs) in cell division, beyond their well-characterized functions in chromatin regulation during cell differentiation and development. In the last two  decades, diverse experimental evidence has shown that subunits of SRCAP and p400/TIP60 chromatin remodeling complexes in humans relocate from interphase nuclei to centrosomes, spindle or midbody, with their depletion yielding an array of aberrant outcomes of mitosis and cytokinesis. Remarkably, this behavior is shared by orthologous subunits of the *Drosophila melanogaster* DOM/TIP60 complex, despite fruit flies and humans diverged over 700 million years ago. In short, the available data support the view that subunits of these complexes are a new class of moonlighting proteins, in that they lead a "double life": during the interphase, they function in chromatin regulation within the nucleus, but as the cell progresses through mitosis, they interact with established mitotic factors, thus becoming integral components of the cell division apparatus.  By doing so, they contribute to ensuring the correct distribution of chromosomes in the two daughter cells and, when dysfunctional, can cause genomic instability, a condition that can trigger tumorigenesis and developmental diseases. Research over the past few years has unveiled a major contribution of long non-coding RNAs (lncRNAs) in the epigenetics regulation of gene expression which also impacts on cell division control. Here, we focus on possible structural roles of lncRNAs in the execution of cytokinesis: in particular, we suggest that specific classes of lncRNAs relocate to the midbody to form an architectural scaffold ensuring its proper assembly and function during abscission. Drawing attention to experimental evidence for non-canonical extranuclear roles of chromatin factors and lncRNAs has direct implications on important and novel aspects concerning both the epigenetic regulation and the evolutionary dynamics of cell division with a significant impact on differentiation, development, and diseases.

## Introduction

**Fig. 1:**
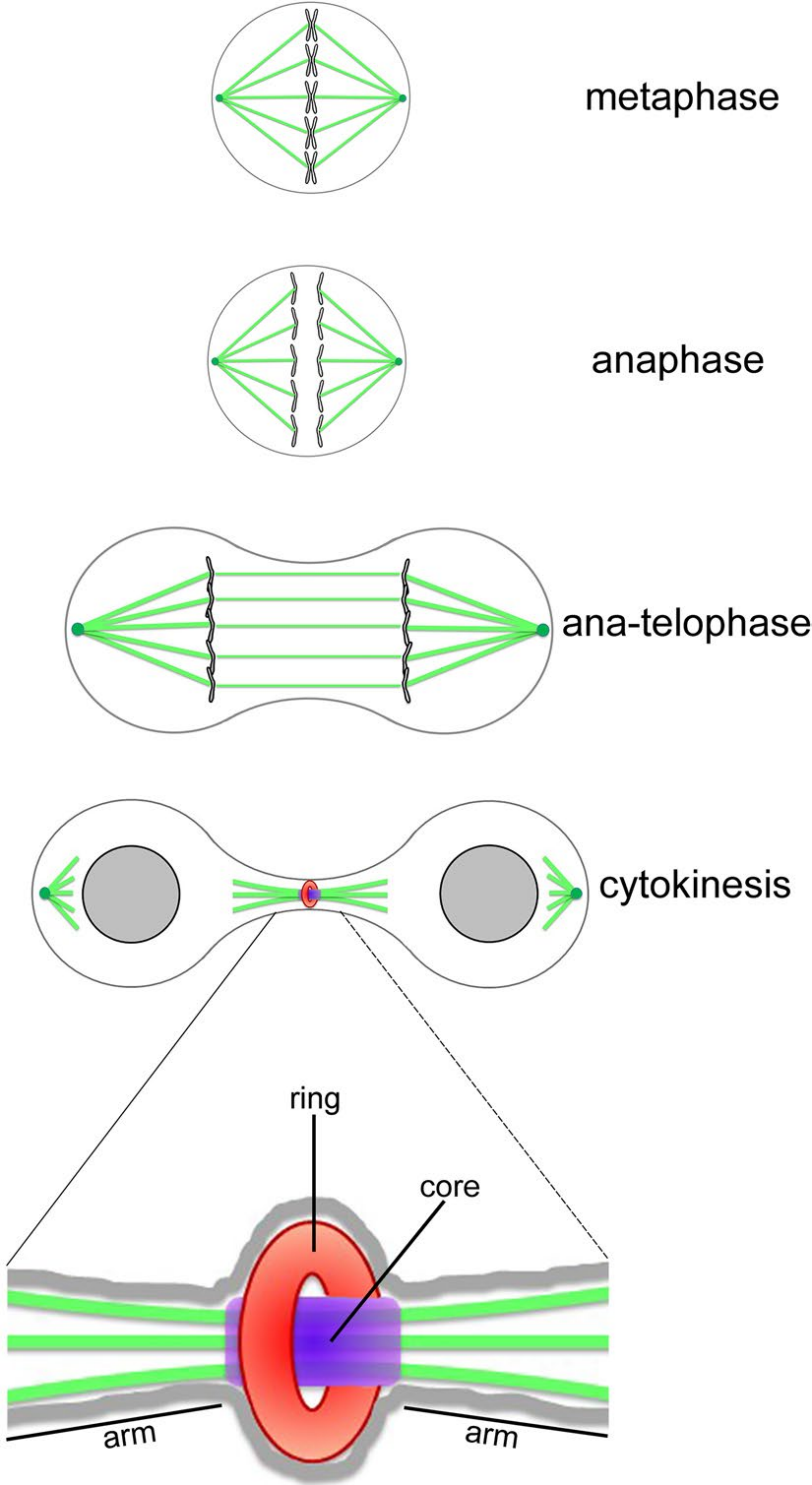
Schematic representation illustrating various stages of cell division and highlighting the three major structural regions of the MB: the ring, central body, and arm

In eukaryotes, successful cell division requires the proper distribution of chromosomes and cytoplasmic material to daughter cells, orchestrated via coordinated cytoskeletal processes including spindle assembly, spindle positioning, chromosome segregation, and cytokinesis [1–9]. Upon entering mitosis, chromosomes condense and attach to mitotic spindle fibers to ensure that sister chromatids are pulled towards opposite sides of the cell (Fig. 1). The mitotic spindle assembles from microtubule arrays and associated proteins that orchestrate chromosome segregation during mitosis [3, 4]. The spindle is highly dynamic in nature and evolutionarily conserved, with many components shared by humans and simpler organisms. In addition to tubulins, proteins involved in spindle function include motor proteins, microtubule-associated proteins (MAPs), microtubule organizing centers, regulatory kinases and phosphatases, kinetochore protein complexes, and chromatin-associated proteins. Following chromosome segregation, the assembly of actomyosin contractile ring occurs at the cleavage furrow. The ring drives the constriction of the plasma membrane leading to abscission, the last stage of cytokinesis (Fig. 1). Before the final cut, the two newly generated daughter cells remain connected by a cytoplasmic bridge that contains the midbody (MB), an organelle first described by Walther Flemming at the end of the 1800s [5]. The MB is a complex multi-protein organelle with a tightly packed structure that forms from the bipolar microtubule array of the central spindle. It plays a pivotal role in the final step of cell division by localizing the site of abscission, and hence of physical separation of daughter cells during cytokinesis [6–12]. Several cellular and molecular pathways have been identified that localize to the MB, contributing to the proper execution of cytokinesis. Notably, various biochemical assays on MB-purified extracts have identified proteins not only related to the cytoskeleton, but also to other molecular pathways, including lipid rafts, vesicle trafficking, protein synthesis, and chromatin organization [6, 10].

The MB consists of three major structural regions: the ring, central core, and arms (Fig. 1) [10]. MB-proteins are generally classified in subgroups based on their location: the ring contains Anillin, Citron kinase, and other contractile ring components; the central core is marked by central spindle proteins, e.g. the Centralspindlin complex; the arms contain the Aurora B kinase and its localizing partners of the Chromosome Passenger Complex (CPC).

Studies on mitosis and cytokinesis are increasingly relevant to cancer research. Anomalies in the mitotic spindle can impact chromosome segregation, leading to aneuploidy. This phenomenon results in chromosomal instability, a significant contributor to genetic heterogeneity in cancer. Additionally, chromosomal instability plays a crucial role in clinical prognosis and the development of therapeutic resistance [13–16]. Furthermore, MB alterations can lead to cytokinesis failure, resulting in two outcomes: (i) inhibition or regression of the cleavage furrow, leading to the formation of binucleated cells, or (ii) persistent connections between daughter cells, forming long intercellular bridges and giving rise to syncytial cells. Cytokinesis failure ultimately yields tetraploid and polyploid cells with multiple centrosomes, which can further result in aneuploid daughter cells. All these dysfunctions converge to promote tumorigenic transformation [16–18]. Consequently, understanding the molecular mechanisms underlying mitosis and cytokinesis holds the potential to significantly impact both cancer prognosis and therapy.

## ATP-dependent chromatin remodeling complexes

Chromatin organization and remodeling are crucial aspects of development and differentiation of higher eukaryotes. In this context, ATP-dependent chromatin remodeling complexes are multi-protein machines that have been highly conserved during eukaryotic genome evolution [31]. These complexes use the energy from ATP hydrolysis to control sliding and displacement of the nucleosomes, thereby modulating histone-DNA interactions and making nucleosomal DNA more accessible to specific binding proteins during replication, transcription, and DNA repair, processes that are crucial for the proper execution of cell division.

Currently, chromatin remodeling complexes are categorized into four subfamilies based on their associated ATPase subunits [19]: (i) the mammalian switch/sucrose non-fermenting (SWI/SNF) subfamily, also called BAF complexes (Brg/Brm-associated factor); (ii) the chromodomain helicase DNA-binding (CHD) subfamily; (iii) the imitation switch (ISWI) subfamily; and (iv) the inositol requiring 80 (INO80) subfamily, which includes yeast INO80 and SWR1 complexes, as well as human p400/TIP60 and SRCAP complexes.

**Fig. 2:**
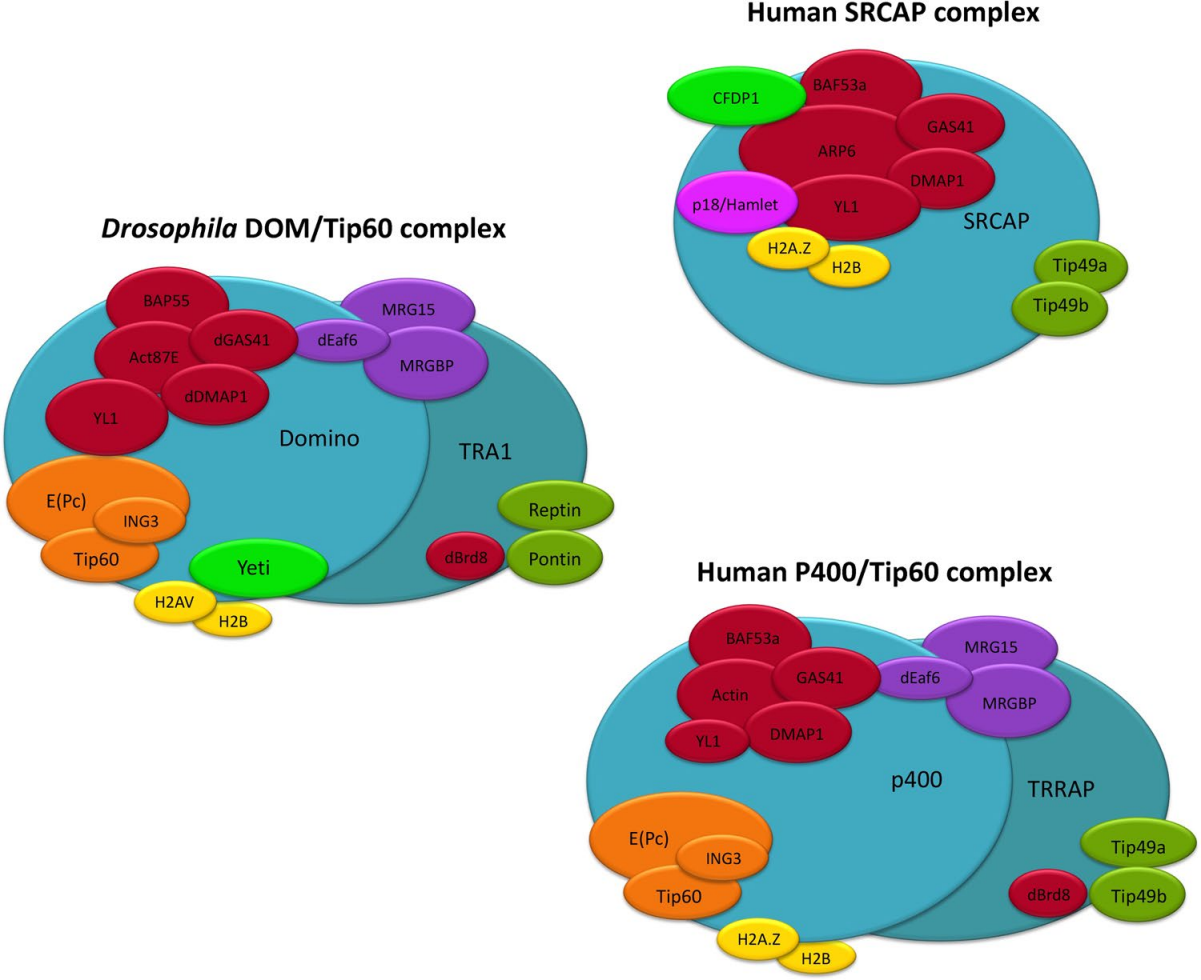
The cartoon shows the subunit composition of *D. melanogaster* DOM/Tip60 and related human SRCAP and p400/Tip60 complexes. The DOM/Tip60 complex consists of 17 known subunits (Act87B, BAP55, Brd8, DOM, DMAP1, E(Pc), Eaf6, GAS41, Ing3, Mrg15, MrgBP, Nipped-A, Pontin, Reptin, TRA1, YETI, and YL1). This complex is crucial for the replacement of acetylated phospho-H2A.V with unmodified H2A.V through the activity of Domino ATPase. In *Drosophila*, H2A.V is the only H2A variant and corresponds to mammalian H2A.X and H2A.Z [20]. Subunits are not in scale

The INO80 family is responsible for exchanging the canonical histone H2A with the variant H2A.Z in various eukaryotic species [19]. The *Drosophila* DOM/TIP60, related to the yeast SWR1 complex shares many subunits with the p400/TIP60 and SRCAP complexes. It has been recently suggested that the subunits assigned to *Drosophila* DOM/Tip60 complex are indeed part of two different chromatin remodeling complexes, DOM-A and DOM-B. These complexes are analogous to the yeast SWR1 and NuA4 complexes, respectively, and are characterized by different functions and subunit compositions [20]. Overall, the subunits of these remodeling complexes exhibit strong evolutionary and functional conservation (Fig. 2; Table 1) and their dysfunction is implicated in cell cycle alterations and tumorigenesis (Table 2).

**Table 1:** Evolutionary conservation of ATP-dependent chromatin remodeling complexes

Organism	*H. sapiens*		*D. melanogaster*	*S. cerevisiae*		*A. thaliana*	
Complex	p400/Tip60	SRCAP	DOM / Tip60	NuA4	SWR1	AtNuA4	AtSWR1
Core	P400	SRCAP	DOM-A	EAF1	SWR1	EAF1A / EAF1B	PIE1
	RUVBL1		Pontin		RVB1		TIP49a (RIN1)
	RUVBL2		Reptin		RVB2		RVB2A / RVB2B
	BAF53a (ACTL6A)		BAP55	ARP4		ARP4	
	YEATS4		GAS41	YAF9		YAF9A / YAF9B	
	DMAP1		DMAP1	SWC4 (EAF2)		SWC4	
	VPS72		YL1		SWC2		SWC2
	Actin		Act87E	ACT1		ACT1	
		ACTR6	ARP6		ARP6		ARP6
	Tip60 (KAT5)		Tip60	ESA1		HAM1 / HAM2	
	MORF4L1		MRG15	EAF3		MRG1 / MRG2	
	MEAF6		dEAF6	EAF6		EAF6	
	MRGBP		MRGBP	EAF7		EAF7	
	EPC1		E(Pc)	EPL1		EPL1A / EPL1B	
	ING3		ING3	YNG2		ING1 / ING2	
	TRRAP		Nipped-A (dTra1)	TRA1		TRA1 / TRA2	
	BRD8		Brd8		Bdf1		
		ZNHIT1			SWC6 (VPS71)		SEF
		CFDP1	YETI		SWC5		SWC5
Non-homologous subunits				EAF5	SWC3, SWC7		

The *D. melanogaster* DOM/TIP60 complex appears to be a fusion of SWR1 and NuA4 complexes of yeast, since the TIP60 complex shares conserved subunits with either SWR1 or NuA4 complexes. Similarly, the human TIP60 complex is a fusion of SWR1 and NuA4 complexes

**Table 2:** Subunits of SRCAP and p400/TIP60 complexes implicated in cancer biology

Subunits	Implication in cancer biology
BAF53a	Overexpressed in bladder, cervix, myeloma, colon, and ovarian cancers [21]; aberrant expression correlated with the progression of rhabdomyosarcoma, osteosarcoma, hepatocellular carcinoma, and head and neck squamous cell carcinoma [21]; BAF53a is considered to promote cancer progression via EMT epithelial–mesenchymal transition [22]
EPC1	Is mutated by chromosomal translocation in endometrial stromal sarcoma and in adult T-cell leukaemia/lymphoma SO4 cells [23–25]
GAS41	Overexpressed in glioblastoma cell lines [26, 27]
MRGBP/MRGX/MRG15	Overexpressed in colorectal cancer tissues [28]
P400	Its siRNA-mediated decrease favors the response to 5-fluorouracil of colon cancer cells [29]
Pontin & Reptin	Overexpressed in bladder, colon, liver cancers, and melanoma [21, 30, 31]
SRCAP	Mutated in large intestine, cervix, bone, endometrium, lung, urinary tract [21]; overexpressed in pancreatic cancer [21]; interacts with the CREB binding protein (CBP), an acetyltransferase encoded by a haplo-insufficient tumor suppressor gene in B-cell lymphoma [32]. It has been identified as a physiologically relevant mediator of PSA expression in prostate cancer cells [33]
Tip60	Acts as a haplo-insufficient tumor suppressor [29, 34]
YL1	Overexpressed in prostate cancer [35]

## Relocation and functions of chromatin factors during cell cycle progression

The first example of versatile chromosome proteins able to change their localizations and functions during cell-cycle progression is given by the CPC, whose subunits are Aurora B, INCENP, Borealin, and Survivin [36]. In early mitosis, they associate with chromosomes, then are recruited to kinetochores to monitor their interactions with the spindle microtubules and eventually relocate to the MB. The catalytic component Aurora B, whose localization is aided by the three passenger partners, phosphorylates and activates several factors at specific times at these different locations, thus playing essential roles in cell division.

The dynamic behavior during cell-cycle progression is not restricted to the CPC proteins. In fact, diverse chromatin proteins, in addition to their role in modulating chromatin organization and gene expression, can relocate to centrosomes, spindle and MB, taking part in cell division and hence in the maintenance of genomic integrity and stability (Table 3). The chromatin proteins Skeletor and Chromator, interact with each other and redistribute during mitosis to form a molecular spindle matrix complex [37, 38]. The Nucleolar and Spindle-Associated Protein (NuSAP), a RanGTP-dependent microtubule and DNA-binding protein, is nucleolar during interphase, associates with microtubules in metaphase and with the central spindle in anaphase and its depletion results in mitosis and cytokinesis defects [39, 40]. In addition, the chromatin remodeler INO80 and three subunits of the SRCAP and p400/TIP60 chromatin remodeling complexes, Pontin (RUVBL1), Reptin (RUVBL2) and TIP60 (KAT5), were shown to relocate to the mitotic apparatus with functional relevance in ensuring the proper execution of cell division in human cells [41–48]. Notably, these proteins were found to interact with tubulins and/or with cell division regulators. INO80 binds to microtubule and was implicated in spindle assembly [41]. Pontin associates with the mitotic spindle and centrosomes via interactions with tubulins in U937 human promonocytic cells [42] and interacts with the γ-tubulin ring complex in *Xenopus* egg extracts [43]. Pontin and Reptin were found on the mitotic spindle [43]. In late anaphase, both Pontin and Reptin concentrate at the MB in HeLa cells [44, 45]. Accordingly, both Pontin and Reptin are found in microtubule interactome [46], and their RNAi-depletion leads to cell cycle alterations such as spindles defects, misaligned, and lagging chromosomes [43–45]. The TIP60 acetyltransferase of the p400/TIP60 chromatin remodeling complex co-localizes and physically interacts with both Plk1, a mitotic kinase, and cyclin B1, forming a ternary complex that localizes to the centrosomes and to the MB during cytokinesis [47]. Moreover, TIP60 performs Aurora B acetylation at kinetochores and is required for proper chromosome segregation [48]. Finally, an RNAi screening in *Drosophila melanogaster* S2 cells provided evidence for a role in cytokinesis of BAP55 [49], a subunit of the *Drosophila* DOM/TIP60 complex (Fig. 2; Table 1).

**Table 3:** Localization of chromatin factors to sites of cell division apparatus, in human and *D. melanogaster* cell cultures

*Homo sapiens*		
Name	Localization	References
Aurora B	Kinetochores, spindle midzone, midbody	[36]
INCENP	Inner centromeres, spindle midzone, equatorial cortex	[36]
Borealin	Centromeres, spindle midzone, cleavage furrow	[36]
Survivin	Centromeres, spindle midzone, cleavage furrow	[36]
NuSAP	Central spindle	[39, 40]
Ino80	Mitotic spindle	[41]
Pontin, Reptin	Mitotic spindle, centrosomes, midbody	[42, 44–46, 51]
BAF53a, CFDP1, GAS41, YL1	Mitotic spindle, central spindle, midbody	[51]
p400	Midbody	[51]
SRCAP	Centrosomes, mitotic spindle, midbody	[50]
TIP60 (KAT5)	Spindle poles, kinetochores, cleavage furrow, central spindle, midbody	[47, 48, 51]

*Drosophila melanogaster*		
Skeletor	Skeletor defined spindle, microtubule spindle	[37]
Chromator	Chromator defined spindle, centrosomes	[38]
dTIP60	Spindle, centrosomes, midbody	[51, 52]
DOM-A	Centrosomes; midbody	[50–52]
MRG15	Centrosomes; midbody	[51, 52]
YETI	Spindle, midbody	[51, 52]
BAP55	Spindle, centrosomes	[52]
DMAP1	Centrosomes	[52]
YL1	Centrosomes	[52]

**Fig. 3:**
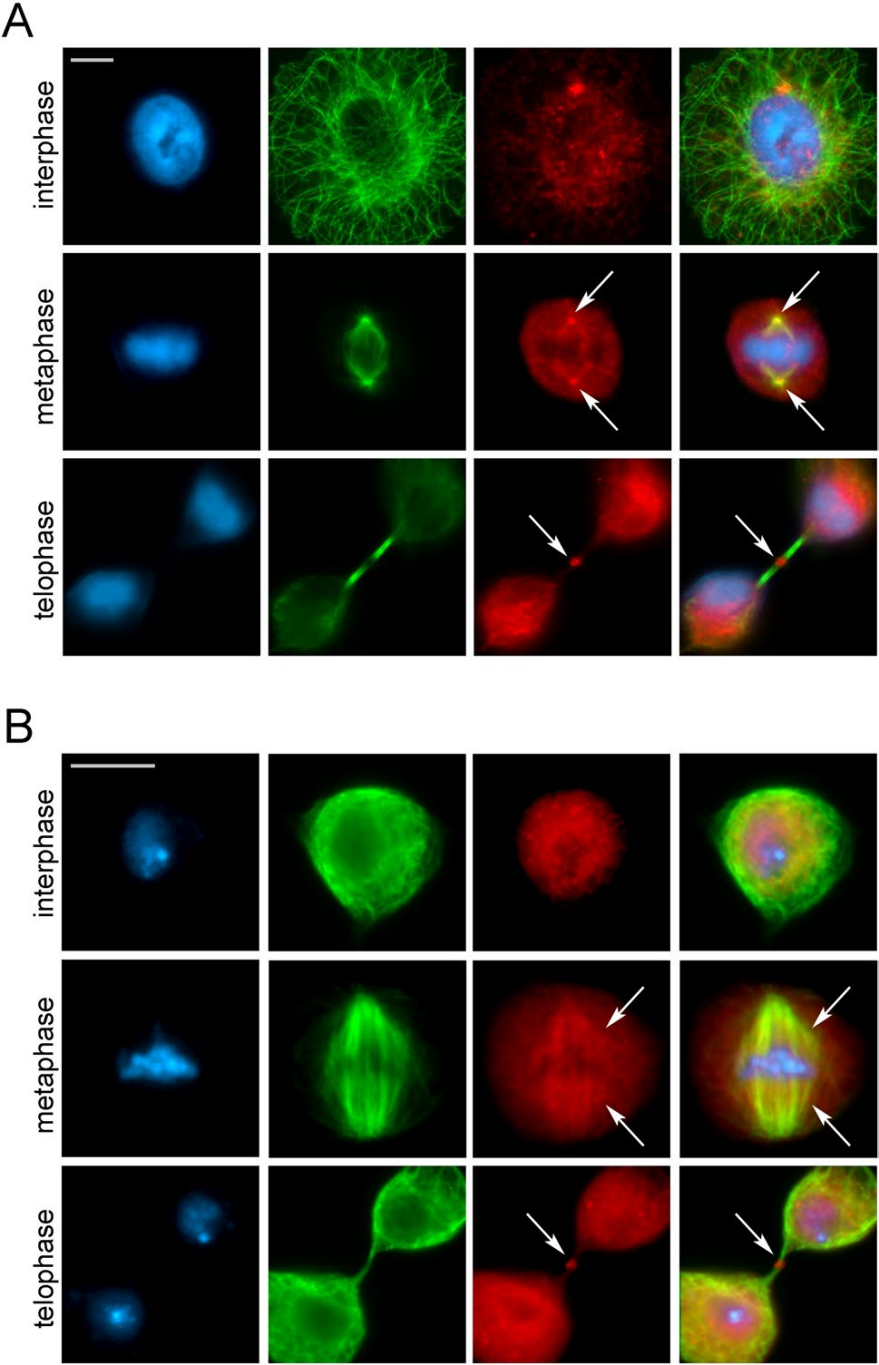
Evolutionarily conserved relocation of chromatin remodelers to the cell division apparatus: the example of TIP60 acetyl-transferase. Immunofluorescence images depict the conserved relocation of chromatin remodelers to the cell division apparatus. Human RPE-1 (**A**) and* D. melanogaster* S2 cells (**B**) were  stained with DAPI (blue), anti-α-tubulin (green), and anti-TIP60 (red). Arrows highlight the centrosome/spindle structures (metaphase) and midbodies (telophase). Scale bars:10 μm (RPE-1) and 5 μm (S2)

More recently, we studied several subunits of human SRCAP and p400/TIP60 chromatin remodeling complexes and found that they localize at the mitotic apparatus (centrosomes, spindle, and MB) in both HeLa and U2OS cell lines (Table 3; Fig. 3A), with their RNAi-induced depletion producing cell division defects at both mitosis and cytokinesis [50, 51]. These defects might be a secondary effect caused by general chromatin perturbations that in turn would result in deregulation of cell division genes. However, a direct role of chromatin remodeling subunits in cytokinesis is supported by co-IP experiments performed on chromatin-free protein extracts from telophase-synchronized HeLa cell [50, 51]. In these assays, where chromatin contribution can be excluded, SRCAP, BAF53a and TIP60 interacted at telophase with α-tubulin, Aurora B, CIT-K, and other MB-associated cytokinesis regulators. Remarkably, a similar relocation behavior during mitosis and meiosis is shared by subunits of DOM/TIP60 complex of *D. melanogaster* (Table 3; Fig. 3B), which are orthologous to those of SRCAP and p400/TIP60 human complexes (Table 1) [51, 52]. Since the lineages of *D. melanogaster* and humans separated approximately 780 million years ago, these results strongly suggested that the functional recruitment of chromatin remodelers to the mitotic apparatus is a very ancient and biologically functional process [51].

Several ATPases interact with microtubules and play direct roles in mitosis and cytokinesis. Specifically, the AAA-ATPase Cdc48/p97 regulates spindle disassembly at the end of mitosis [53]. The ISWI activity is necessary for spindle maintenance, stabilizing microtubules in anaphase [54], while INO80 and CHD4 are required for spindle microtubules assembly [41, 55]. Interestingly, domain analysis of ISWI, CHD4, and INO80 revealed that they can bind microtubules through regions containing chromatin-binding domains [56]. Moreover, Spastin, a microtubule-severing ATPase ensuring the final cut at the midbody, assembles into a hexamer and recognize the C-terminal amino acids of α-tubulin [57]. Mutations in the Spastin-encoding gene are the most common causes of dominant hereditary spastic paraplegias (HSPs), a genetic motor neuron disease characterized by progressive degeneration of corticospinal tract axons [58]. Interestingly, depletion of Spastin in HeLa and MRC5 cells results in cytokinesis failure phenotypes [59], similar to those found in SRCAP-depleted HeLa cells [50]. In agreement with these results, the SRCAP ATPase interacts with Spastin and α-tubulin [50] in telophase-synchronized cells and carries putative microtubule-binding domains in its C-terminal region (Y. Prozzillo, unpublished). Thus, we hypothesized that SRCAP ATPase, as spindle and MB component, participates in two different steps of cell division: by ensuring proper chromosome segregation during mitosis and MB function during abscission at cytokinesis [50].

Dominant truncating mutations of the *Srcap* gene were found to trigger the onset of Floating Harbor syndrome (FHS), a rare genetic disease characterized by delayed bone mineralization and growth, skeletal, and craniofacial abnormalities, often associated with mental disability [60, 61]. Thus, in addition to gene deregulation caused by chromatin alterations, a cell division failure may contribute to the developmental defects found in FHS patients [50].

Collectively, the aforementioned studies highlighted the existence of a massive and evolutionarily conserved phenomenon where relocation of chromatin factors to the cell division apparatus has functional relevance in ensuring a faithful cell division in distantly related organisms. It is possible to speculate that the “mitotic trip” of chromatin remodelers from the interphase nucleus to the cell division apparatus takes place by exploiting interactions with microtubules and/or microtubule-associated proteins. Elucidating the molecular mechanisms underlying the moonlighting functions of chromatin proteins in cell division is also an important challenge to clarify yet poorly understood routes to tumorigenic transformation in cancer types in which these factors are aberrantly expressed and/or dysfunctional (Table 2). Gaining insight into these processes will also help to expand our understanding of the link between cell division and cancer.

## Chromatin remodeling and lncRNAs

LncRNAs are crucial players controlling a plethora of biological processes [62, 64] and their deregulation is also implicated in tumorigenesis [63–66]. As the list of cancers aberrantly expressing lncRNAs is growing fast, lncRNAs have been proposed both as novel biomarkers and potential therapeutic targets for cancer [65, 66]. Increasing evidence shows that many lncRNAs are involved in chromatin regulation and gene expression and can function as scaffolds for the recruitment of chromatin factors [63]. Several lncRNAs facilitate the binding and spreading of the Polycomb repressive complex 2 (PRC2) across targeted chromatin [67–69]. Interactions between lncRNAs and subunits of different chromatin remodeling complexes such as BAF, SRCAP, NuRD, and ATRX complexes have been reported [70]. For example, the lncRNA SChLAP1 interacts with SNF5, a core subunit of the BAF complex, which is required for the proper assembly and function of the complex [71]. The well-known lncRNA XIST physically associates with the BRG1 subunit of the BAF complex and inhibits its ATPase activity in vitro [72]. The lncRNA SWINGN promotes the interaction between SWI/SNF chromatin remodeling complexes and the transcription start site of GAS6 oncogene [73]. The SWI/SNF complexes have been also identified as paraspeckle components that interact with the lncRNA NEAT1 [74]. Remarkably, the lncKdm2b, a highly expressed lncRNA in murine embryonic stem cells, interacts with SRCAP, the main subunit of the homonymous complex increasing its ATPase activity [75]. This finding is of particular interest in light of the roles played by SRCAP in mitosis and cytokinesis [50].

## Regulatory roles of RNAs in cell division

Over the last two decades, several studies emerged supporting the regulatory roles of RNAs, both mRNA and lncRNAs, during cell division in evolutionary distant organisms. Evidence for non-canonical localizations of mRNAs to the mitotic apparatus suggested that they could be involved in the regulation of cell division. Centrosomally localized mRNAs were found in embryos of the mollusc *Ilyanassa obsoleta* [76] and in oocytes of the surf clam *Spisula solidissima* [77]. In *Xenopus laevis* egg extracts, mitotic spindle-associated RNA has been identified and suggested to play a translation-independent role in spindle assembly [78]. In mitotic extracts of both *X. laevis* and humans, a significant fraction of mRNAs was also identified that targets microtubule during mitosis, suggesting a conserved mechanism to regulate mitotic events and delivering translationally inactive mRNAs to daughter cells [79]. In early stages of *D. melanogaster* embryogenesis, several mRNAs were found associated with spindle poles, centrosomes, astral microtubules or the mitotic spindle [80], indicating that mRNA localization may play a key role in targeting various cellular machineries, including those involved in protein synthesis. The mRNA localization to subcellular structures was originally found to occur through the 3ʹ UTR regions, such as those of *nanos* and *bicoid* in early *Drosophila* embryos [81–83]. These and other findings [84–87] also provided evidence for a mitotic apparatus localized mRNA translation, whose initial concept emerged in the late 1950s and early 1960s [88]. Very recently, evidence have been provided showing a localized enrichment and translation of midbody associated mRNAs encoding key regulatory factors of cytokinesis [89, 90]. Remarkably, the results of Park et al. [89] suggested that the mitotic kinesin MKLP1 and the actively regulated cytoskeleton-associated protein, ARC, are necessary for the localization and translation of mRNAs in the MB dark zone, while ESCRT-III, a protein normally required for the abscission process, maintains translation levels in the MB.

Regulatory roles of lncRNAs in controlling cell division are increasingly being demonstrated [91]. A high-content RNAi imaging screen targeting more than 2,000 human lncRNAs yielded the identification of numerous lncRNAs controlling mitotic progression, chromosome segregation and cytokinesis via regulation of key cell division players [92]. A regulatory lncRNA, termed mamRNA, was shown to be a crucial player in shaping the meiotic gene expression program in fission yeast by scaffolding the antagonistic RNA-binding proteins Mmi1 and Mei2 [93]. More recently, a widely expressed circular RNA, circZNF609, interacts with several mRNAs and increases their stability and/or translation by facilitating the recruitment of the RNA-binding protein ELAVL1 [94]. In particular, circZNF609 interacts with CKAP5 mRNA, which encodes a microtubule-stabilizing factors, and enhances its translation, thus regulating microtubule function and sustaining cell cycle progression. Importantly, circZNF609 also modulates the cancer cell response to microtubule inhibitors used in cancer chemotherapy [94].

## LncRNAs as architectural components of the midbody: a working hypothesis

A large fraction of lncRNAs is exported to the cytosol, where they could be assigned to specific organelles or distributed in the cytoplasm associating with RNA-binding proteins [63]. Moreover, lncRNAs are emerging players functioning as phase separation anchors in different subcellular localizations and in the formation of biomolecular condensates [95–98]. Well-known examples are given by NEAT1 and NORAD lncRNAs [98–100].

In light of these functions, it is an intriguing possibility that ncRNAs, in addition to their well-established regulatory functions in controlling of cell division, also play structural roles at sites of the mitotic apparatus [101]. A direct physical role of a human centromeric 1.3 kb long lncRNA in maintaining centromere integrity has been proposed [102]. By targeting CENP-A and its chaperone HJURP to the centromere, this lncRNA ensures proper chromosome dynamics during mitosis and its knockdown results in the formation of multipolar spindles and lagging chromosomes [102].

Several cellular and molecular pathways and categories of proteins have been assigned to the MB, contributing to the proper execution of cytokinesis (Fig. 1) [6, 10]. Moreover, MB-associated mRNAs have been recently reported [89, 90]  but  little is known about the presence of architectural lncRNAs on this organelle.

**Fig. 4:**
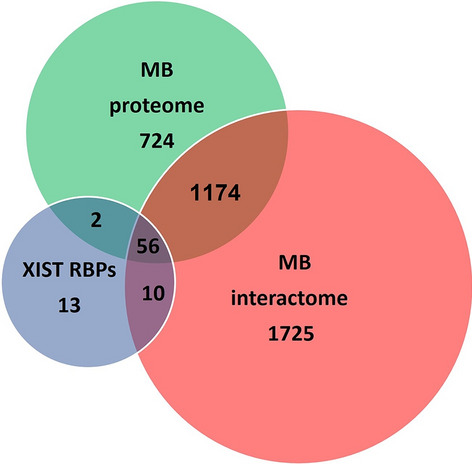
Venn diagrams illustrating overlapping protein sets. The Venn diagrams present an analysis of protein sets to reveal commonalities between mouse XIST-binding proteins [106] and orthologous proteins detected in the human MB-proteome and interactome. The overlapping between circles shows the number of proteins in common between the groups. Notably, among the 81 XIST RNA-binding proteins found in mouse, 56 have orthologs in both the human proteome and the interactome of MB. [Y. Prozzillo, unpublished]

So far, only few examples suggesting a recruitment of lncRNAs to the MB have been reported. First, Moulton Clemson et al., using FISH, described MB localization of XIST RNA during cytokinesis in human female fibroblasts [103]. XIST RNA, the first long non-coding RNA to be identified, is a major epigenetic effector triggering the X-chromosome inactivation in mammalian female cells [104]. This result would also imply a role of XIST in female cell division which was not further investigated. Intriguingly, the Aurora B kinase, a key component of the MB, interacts with XIST RNA controlling its binding to chromatin [105]. However, the FISH signals found with the XIST probe at the cleavage plane [103] appeared to be somewhat dispersed and not precisely aligned with the MB/central spindle structure. Recently, Chu et al., have identified 81 XIST RNA-binding proteins in mouse cell lines [106]. Interestingly, we discovered that 56 orthologs of these XIST RNA-binding proteins are present in the human MB proteome and interactome datasets (Fig. 4) [10]. These findings collectively provide support for the recruitment of XIST at MB. Finally, in mouse 3T3 cells, the GAA repeat-containing RNAs (GRC-RNAs), a polypurine triplet repeat-rich lncRNA, was found to localize at the midzone area in early telophase and at the MB in late telophase [107]. Finally, the lncKdm2b interacts with the SRCAP ATPase [75], which is recruited to spindle and MB in HeLa and MRC5 cells [51]. However, it is unknown whether the interaction between lncKdm2b and SRCAP is nucleus-specific or also occurs at the MB.

**Fig. 5:**
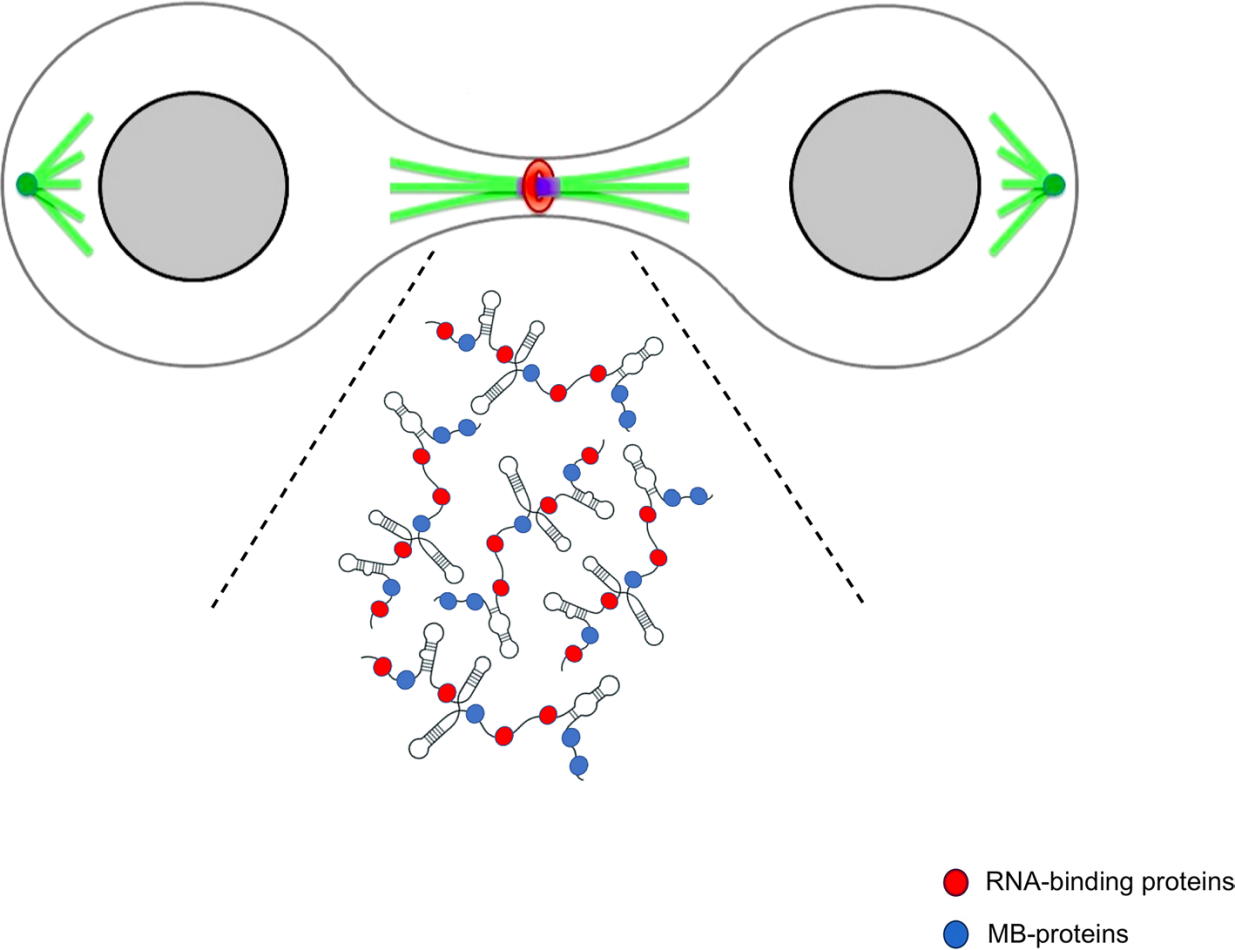
LncRNA–MB-proteins aggregates triggering MB assembly. LncRNAs promote the formation of phase separation, functioning as architectural scaffolds for diverse RNAs and proteins interaction giving rise to biomolecular condensates in different subcellular localizations [95–98]. The cartoon shows a hypothetical network of interactions between lncRNAs and proteins (RNA-binding proteins, MB-proteins including cytokinesis regulators, chromatin remodelers, and other MB-associated factors) driving the formation of molecular aggregates that trigger the proper MB assembly during cytokinesis

Together, the sparse evidence recalled above hints at the fascinating possibility that lncRNAs serve as structural components of the MB. In other words, specific classes of lncRNAs might have the ability to interact with RNA-binding proteins, cytokinesis regulators and other factors, providing an architectural platform for MB assembly (Fig. 5), thus contributing to the proper execution of cytokinesis. Since MB dysfunctions cause abscission failure, leading to genetically unstable states that would promote tumorigenic transformation, investigating in depth the roles of lncRNAs in MB assembly and function can have a strong impact on cancer biology.

## Conclusions: an upcoming challenge

To comprehensively explore and dissect the phase-specific roles of epigenetic players in cell division, their degradation could be performed in a time-controlled way using different approaches. One effective method is the immuno-depletion technique originally developed by Gergely et al., [108], which allows for the inactivation of a given protein of interest (POI) through the injection of specific interfering antibody. This approach has recently proven successful in providing evidence for the mitotic roles of the splicing factors Sf3A2 and Prp31 in *Drosophila melanogaster* embryos [109]. Over the years, several tools have been devised to achieve protein degradation via proteasome recruitment. Among those, the PROTACs (PROteolysis Targeting Chimeras) systems rely on chimeric molecules composed of a specific ligand for the POI and an E3 ubiquitin ligase. Advances of this system include the use of light-responsive degraders with photocaged or photoswitchable molecules [110]. Targeted protein inactivation can be also achieved by the adding a specific tag fused with the POI. In such cases, a small heterobifunctional molecule can bind the tagged protein, inducing its inactivation through dysfunctional mislocalization or proteosomal degradation [111]. These systems have been widely employed to dissect protein functions in diverse model systems [112, 113].

The dynamic relocation of lncRNAs during cell division can be examined using the *CasFAS* system, a recently developed imaging method for visualization of endogenous RNAs in living cells [114]. Additionally, optogenetics systems based on light-responsive molecules can be refined and integrated with other techniques, such as the recombinant codon-optimized Cas9 (rCas9), to provide valuable tools for investigating potential cell division phase-specific structural roles of lncRNAs [115–119].

In conclusion, unraveling the unexpected roles of epigenetic regulators during mitosis and cytokinesis in different organisms presents a significant challenge in the field of cell biology that can be met through the combined application of the aforementioned approaches.

## Data Availability

The datasets generated during the current study are available from the corresponding author on reasonable request.
